# Heterochronies and allometries in the evolution of the hominid cranium: a morphometric approach using classical anthropometric variables

**DOI:** 10.7717/peerj.13991

**Published:** 2022-08-25

**Authors:** Juan Antonio Pérez-Claros, Paul Palmqvist

**Affiliations:** Departamento de Ecología y Geología, Universidad de Málaga, Málaga, Spain

**Keywords:** Heterochrony, Allometry, Human evolution, Hominins, Cranium, Ontogenetic scaling, *Homo naledi*, *Homo longi*, *Australopithecus prometheus*, *Australopithecus anamensis*

## Abstract

This article studies the evolutionary change of allometries in the relative size of the two main cranial modules (neurocranium and splanchnocranium) in the five living hominid species and a diverse sample of extinct hominins. We use six standard craniometric variables as proxies for the length, width and height of each cranial module. Factor analysis and two-block partial least squares (2B-PLS) show that the great apes and modern humans share a pervasive negative ontogenetic allometry in the neurocranium and a positive one in the splanchnocranium. This developmental constraint makes it possible to interpret the cranial heterochronies in terms of ontogenetic scaling processes (*i.e*., extensions or truncations of the ancestral ontogenetic trajectory) and lateral transpositions (*i.e*., parallel translations of the entire trajectory starting from a different shape for a given cranial size). We hypothesize that ontogenetic scaling is the main evolutionary modality in the australopithecines while in the species of *Homo* it is also necessary to apply transpositions. Both types of processes are coordinated in *Homo*, which result in an evolutionary trend toward an increase in brain size and in the degree of paedomorphosis from the earliest habilines.

## Introduction

**Figure 1:**
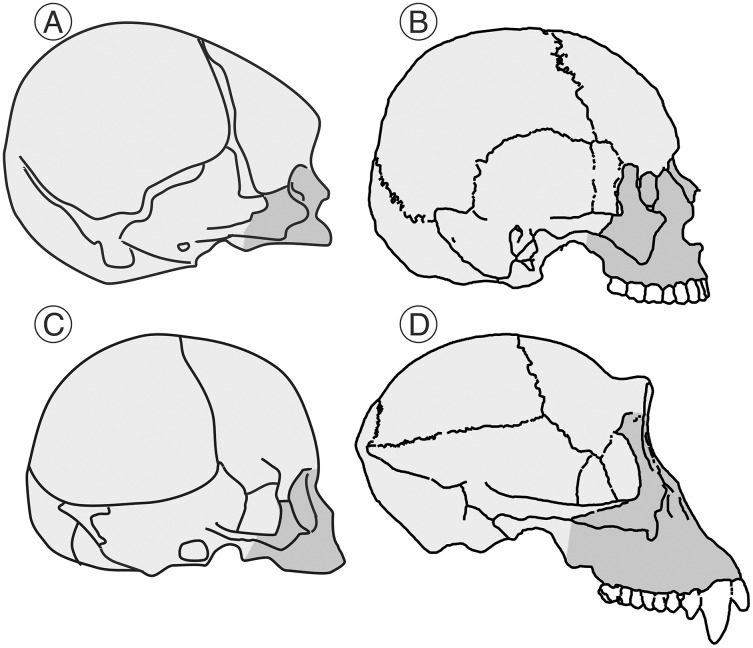
Human and chimpanzee crania. Schematic representation of the cranium of a human neonate (A) and an adult (B) and the corresponding ones for the chimpanzee (C and D, respectively) indicating the approximate size of the splanchnocranium (shaded in dark gray tone) *vs* the neurocranium (shaded in light gray tone). As noted by Gould (1977), the relative size of the neurocranium decreases with growth in both cases, but this decrease is more evident in the case of the chimpanzee.

Heterochronies (shifts in rates or timing of developmental stages or events relative to ancestral patterns) are pervasive in human evolution. Such changes generate much of the raw material on which natural selection works (McNamara, 2012). There are two different types of heterochronies: paedomorphosis (“child-shape”), where the adult state of the descendant would resemble the juvenile condition of the ancestor, and peramorphosis (“beyond-shape”), where the adult of the descendant would show a more extended development compared to the ancestor leading to exaggerated adult traits. The role of heterochrony in human evolution is a classic matter of debate (Shea, 1989; Klingenberg, 1998). In his influential book *“Ontogeny and Phylogeny*”, a landmark in this field, Stephen Jay Gould indicated that humans have a reduced rate of development with respect to their ancestors as a result of ontogenetic delay (Gould, 1977). For Gould this implied a process of developmental retardation, although the two phenomena need not necessarily be linked (for a discussion on this point, see Klingenberg, 1998). In the case of the cranium, the developmental retardation entails both an increase in the absolute size of the brain and a reduction of the face relative to the neurocranium (Fig. 1). In contrast, Shea (1989) criticized Gould’s hypothesis by pointing out that some of the features that he mentioned could be explained by the paedomorphic phenomenon of rate hypomorphosis (earlier developmental onset by decreasing the developmental rate). Shea (1989) further concluded that there is no single heterochronic process that explains human evolution and criticized the lack of experimental evidence in support of the association between extended development and paedomorphosis. McKinney & McNamara (1991) and McKinney (1998) went further in their criticisms, indicating that the increase in the size of the human brain is not a consequence of a paedomorphic process but a peramorphic one that results from hypermorphosis. McNamara (2012) proposed that some features of the human cranium, such as the large brain, are peramorphic while others, such as the reduced face, would be paedomorphic. In conclusion, depending on the structure analyzed, the heterochronic process involved may be interpreted differently by different authors.

Allometry is the covariation between shape and size. Since heterochronies are a source of allometries and allometries evidence heterochronies, both phenomena are conceptually and methodologically related (Klingenberg, 1998). Allometries can be classified in different ways according to the nature of the data analyzed (Klingenberg, 2016). Here we follow the classification of Klingenberg (1998), although there are other methods (*e.g*., Gould, 1966). Within a given species, ontogenetic allometry relates to changes between shape and size along an ontogenetic sequence, while static allometry does so within a specific ontogenetic stage, for example the adult stage (sexual dimorphism falls within this type). Both ontogenetic and static allometry allude to intraspecific covariation. In contrast, for a given developmental stage (usually the adult one), evolutionary allometry analyzes the covariation of size and shape due to the evolutionary change in the species of a clade, regardless of whether there is an ancestor-descendant relationship between them or they are sister species. Empirical studies have shown that the patterns resulting from ontogenetic, static and evolutionary allometry may differ from each other to a greater or lesser degree (*e.g*., Cheverud, 1982a; Klingenberg & Zimmermann, 1992).

**Figure 2:**
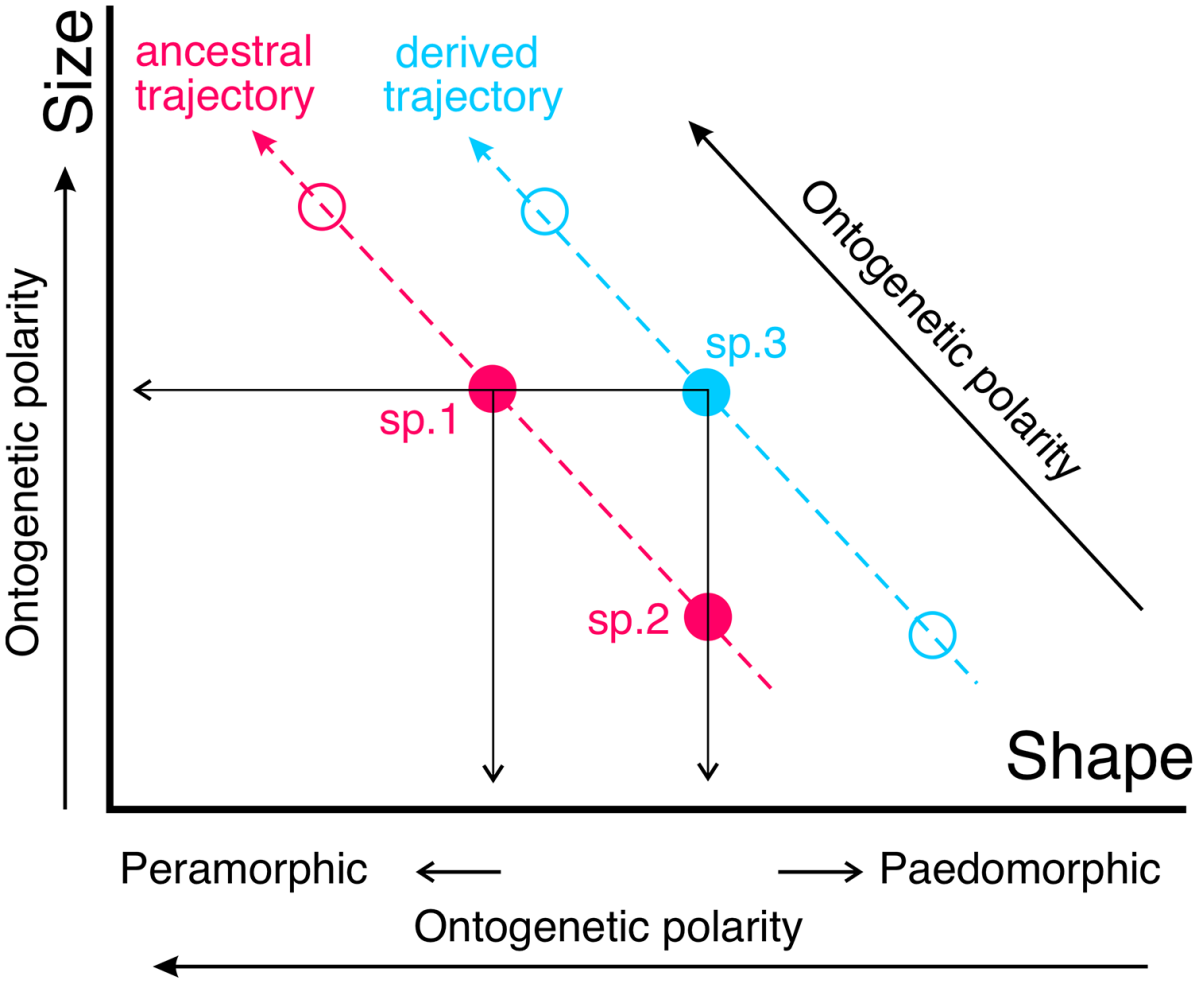
The same type of heterochronies can arise in different ways. Here such a phenomenon is illustrated with two sets of adults from different species belonging to a clade (blue and pink circles, respectively). Species following a given trajectory are related to each other *via* ontogenetic scaling, while lateral transposition relates species following different allometric trajectories. For example, the phenomenon of paedomorphosis can occur in two different ways from an ancestral species (sp.1). In the case of descendant species 2 (sp.2), paedomorphosis is achieved by ontogenetic scaling (through truncation of the ancestral ontogeny), while in the case of descendant species 3 (sp.3) the same result is achieved by lateral transposition. Note that lateral transposition allows in this case a change of shape without altering size, which is not feasible if the derived species follows the ancestral trajectory.

If the ontogenetic polarity of shape change within a given clade is known, it is possible to analyze the general type of heterochronies (*i.e*., paedomorphosis or peramorphosis) in terms of allometries. Ontogenetic scaling is the extension or truncation of a conserved ontogenetic trajectory, which leads to peramorphic forms in the former case and paedomorphic forms in the latter (Fig. 2). In contrast, lateral transposition involves a new ontogenetic trajectory that starts from a different shape for a given size.

Some of the examples used by different authors to illustrate the different types of allometries and heterochronies allude to covariation between parts of the organism that correspond to modules. A module is a part of the organism whose components interact closely with each other, thus forming a functional or developmental entity that is relatively independent of other similar entities (Klingenberg, 2013, 2016; Klingenberg & Marugán-Lobón, 2013; Zelditch & Goswami, 2021). Modules are integrated into larger hierarchies up to the level of the organism. The concept of modularity is becoming a central concept in evolutionary biology (Melo et al., 2016), as the relative autonomy of evolutionary change in some modules with respect to others allows them to adapt to different functions without interfering with others (Wagner & Altenberg, 1996). In the case of the skull, the two most important cranial modules are the neurocranium (*i.e*., the cerebral capsule or neurobasicranial complex) and the splanchnocranium (Cheverud, 1982b; Mitteroecker & Bookstein, 2008), which are inferred from both developmental processes and function (Mitteroecker & Bookstein, 2008).

In the hominids, Pérez-Claros, Jiménez-Arenas & Palmqvist (2015) showed that the relative sizes of the two main cranial modules (neurocranium and splanchnocranium) follow different allometric rules depending on the group considered. The great apes, anatomically modern humans and australopithecines show negative ancestral allometries between both modules. These allometries take the form of ontogenetic scaling within each group. However, at the same time, such sets of hominids are displaced by lateral transpositions from this general pattern of ontogenetic scaling. Lateral transpositions (unlike ontogenetic scaling) imply a dissociation of the growth patterns of both cranial modules (Klingenberg, 1998).

The sample used by Pérez-Claros, Jiménez-Arenas & Palmqvist (2015) only included adult individuals. Consequently, only the static and evolutionary allometries between the two skull modules were studied. In this article, we have extended our original study by including juvenile and infant crania to incorporate ontogenetic allometries of the living hominoid species. Moreover, during the last years, publications of new findings has allowed the incorporation of cranial specimens from potentially four extinct species (Berger et al., 2015; Clarke & Kuman, 2019; Hawks et al., 2017; Haile-Selassie et al., 2019; Ji et al., 2021; Ni et al., 2021): *Australopithecus anamensis*, *A. prometheus*, *Homo naledi*, and *H. longi*. The characterization of the relationships between the relative sizes of both cranial complexes in these newly described species compared to the rest of hominids may be of great interest.

Therefore, the aim of this article is to evaluate the relationships between the ontogenetic and evolutionary allometries in the relative sizes of the neurocranium and the splanchnocranium, in order to characterize the heterochronic relationships between the species studied. Our starting hypothesis is that the developmental constraint of an ancestral negative ontogenetic allometry for the neurocranium and a positive one for the splachnocranium would be shared by all hominids, and impose certain combinations of ontogenetic scaling and lateral transpositions that changed both modules throughout hominin evolution. We hypothesize that the main evolutionary modality in the australopithecines was ontogenetic scaling, while for the species of *Homo* it is also necessary to apply lateral transpositions. Consequently, the different points of view on the role played by heterochrony in human evolution can be reconciled if the level at which any two species are compared is clearly defined.

## Materials and Methods

In this study, we use the methodology described in Pérez-Claros, Jiménez-Arenas & Palmqvist (2015). The size of each cranial module is characterized by three classical anthropometric variables. These measurements, which are contained in orthogonal planes, can be considered as proxies of the length, width and height of each cranial module. In the case of the neurocranium, the variables are glabella-opisthocranion length (GOL), maximum biparietal cranial breadth (XCB) and basion-bregma height (BBH). In the splanchnocranium, we measured basion-prosthion length (BPL), nasion-prosthion height (NPH) and bizygomatic breadth (ZYB). Among primates, much of the diversity in cranial morphology is closely related to the relative development of both cranial modules (Ackermann & Cheverud, 2004). For this reason, the three variables used for estimating the size of each module allow an adequate characterization of the gross dimensions of both cranial complexes, although it must be noted that they do not capture details within them.

The starting database is described in Pérez-Claros, Jiménez-Arenas & Palmqvist (2015) and consists of 291 non-pathological adult specimens of the five living species of hominids and 28 fossil representatives of several hominin species. The number of adult male and female specimens of orangutans and gorillas (*i.e*., the two most dimorphic species) is balanced, consisting of seven females and seven males in the former case and 14 females and 15 males in the latter (see further details in Pérez-Claros, Jiménez-Arenas & Palmqvist, 2015). The sample of fossil crania has been expanded to include specimen MRD-VP-1/1 attributed to *Australopithecus anamensis* (Haile-Selassie et al., 2019), skull StW 573 of *Australopithecus prometheus* (Clarke & Kuman, 2019), the reconstruction of LES1 skull (nicknamed ‘Neo’) of *Homo naledi* (Hawks et al., 2017; Laird et al., 2017), and the newly described cranium HBSM2018-000018(A) attributed to *Homo longi* (Ji et al., 2021; Ni et al., 2021). It is important to emphasize here that we use the original designation attributed to the specimens in the literature from which the metric variables were obtained (*i.e*., we do not attempt to make here any taxonomic inference). For example, *A. prometheus* has been related to *A. africanus* (Berger & Hawks, 2019) and *Homo longi* to the Denisovans (Gibbons, 2021).

In order to consider diverse ontogenetic stages, the original database of Pérez-Claros, Jiménez-Arenas & Palmqvist (2015) has been supplemented with a sample of juvenile and infant individuals of the living hominoid species analyzed: *H. sapiens* (*n* = 9), *Pan troglodytes* (*n* = 10), *Gorilla gorilla* (*n* = 5) and *Pongo pygmaeus* (*n* = 10). Although the sample of *G. gorilla* is undoubtedly very small, which means that the results obtained from it must be considered with caution, it has been kept for comparative purposes. Another limitation of this study is that the age of these specimens is unknown. However, given that cranial size increases monotonically with biological age during the earlier phases of development, the latter tends to be approximated by the former, especially in those studies where fossils are included (Klingenberg, 1998). As discussed by Klingenberg (1998), some authors (*e.g*., Strauss, 1987) prefer to use size as an estimator of biological age rather than age, because the former is better correlated with growth than the latter.

This approach (allometric heterochrony *sensu* Klingenberg, 1998) has been adopted in many studies to interpret heterochronies in terms of allometries (Klingenberg, 1998; Smith, 2001; Vinicius & Lahr, 2003). It is worth noting that this type of study allows a general analysis of heterochronies based on their morphological results (*e.g*., for distinguishing between paedomorphosis and peramorphosis), with independence of the underlying biological processes (Shea, 1989). However, it should be noted that this kind of methodology cannot determine the specific ontogenetic process responsible, since biological age and size are not strictly interchangeable for these purposes (Klingenberg, 1998).

The measurements were taken following the procedure described in Pérez-Claros, Jiménez-Arenas & Palmqvist (2015). Consequently, most of the values used for the craniometric variables of the fossils were obtained from the literature. In those cases where measurements were not published, they were measured on 3D virtual models or photographs. In order to make the measurements obtained from the 3D models compatible with those obtained from photographs, screenshots were taken of the models in the appropriate orientation, from which the measurements used in the analyses were obtained. The differences between the measurements obtained by such a procedure and those taken directly on the 3D models did not exceed 5%. In the case of the hominin fossils analyzed, the average differences between the values of the variables measured from photographs and those provided by the bibliographic sources ranged between 2% and 5%. Moreover, Pérez-Claros, Jiménez-Arenas & Palmqvist (2015) evaluated the robustness of principal component analysis against random variations resulting from inaccuracies of up to 5% in the measurements for those fossils with a lower degree of preservational completeness (*e.g*., cranium SK48 of *Paranthropus robustus*), for which alternative measurements are available in the bibliography (see the Supplemental Information in Pérez-Claros, Jiménez-Arenas & Palmqvist, 2015). The results obtained from 500 simulations indicated that the relative positions of the fossils remained relatively stable, clustering in the principal components around the scores of the original data in all cases, which argues again in favor of the robustness of this study. In those cases in which the zygomatic arches of a cranium were partially missing, they were conservatively reconstructed by joining the preserved part of the zygomatic process of the temporal bone with the zygomatic bone. Similarly, if only one zygomatic arch was present, the measurement ZYB was estimated using the mirror image of the preserved side. All measurements used and their provenances are in Table S1.

The variables were transformed into base 10 logarithms prior to statistical analyses. Two-block partial least squares (2B-PLS) (Rohlf & Corti, 2000), a method traditionally applied for studying cranial morphological integration (*e.g*., Mitteroecker & Bookstein, 2008; Singh et al., 2012; Mitteroecker et al., 2012; Klingenberg, 2013; Pérez-Claros, Jiménez-Arenas & Palmqvist, 2015; Zelditch & Goswami, 2021), was used to study the covariation between both cranial modules. Given that the two cranial modules (blocks) can be indirectly correlated through their correlation with cranial size, 2B-PLS were performed by dividing the six variables taken in each specimen by their geometric mean (Mosimann & James, 1979; Somers, 1989). This size standardization allows us to compare both cranial modules, as each specimen has a geometric mean of 1. As indicated by Pérez-Claros, Jiménez-Arenas & Palmqvist (2015), this method can be considered as an equivalent of the “simultaneous adjustment” approach (*sensu* Klingenberg, 2008) for metric variables, since each variable is scaled to the size of the whole specimen. Variables were not grouped within species to perform 2B-PLS.

Using a similar approach to Pérez-Claros, Jiménez-Arenas & Palmqvist (2015), factor analysis of the log-transformed measurements shown in Table S1 was used to study allometries and heterochronies from a multivariate perspective. Factor and principal component analyses allow us to estimate variation taking into account potential underlying correlations and have been widely used in this type of studies (see review in Klingenberg, 1998) and, as indicated above, have been shown to be robust techniques in the face of small errors in the estimation of variables (Pérez-Claros, Jiménez-Arenas & Palmqvist, 2015). In addition, this methodology is useful for the study of patterns of morphological integration (Lieberman, 2011), which has direct implications for the study of heterochronies (Shea, 1989). Here the factors were obtained directly from principal component analysis by multiplying the eigenvectors by the square root of their corresponding eigenvalues. Reduced major axis regressions were performed on the scores of the two first factors and 2B-PLS.

Clarke’s test (Clarke, 1980) was used for the contrast of differences between RMA slopes. The 2B-PLS was programmed in Wolfram Mathematica (Wolfram Research Inc, 2021) and all other statistical analyses, including the PCA used to obtain the factor analysis, were performed with PAST 3.24 (Hammer, Harper & Ryan, 2001).

## Results

The results obtained with both the 2B-PLS and the factor analysis (Table 1) are nearly identical to those previously shown by Pérez-Claros, Jiménez-Arenas & Palmqvist (2015). Consequently, the inclusion of the new fossils and the sample of juveniles of the extant species did not lead to important changes in the covariation matrices.

**Table 1:** Summary of the 2B-PLS and factor analyses.

		2B-PLS	Factor analysis		
	Variable	Dimension I	Factor I	Factor II	h^2^
Neurocr.	logGOL	0.544	0.673	0.695	0.937
	logBBH	0.597	0.895	0.378	0.944
	logXCB	0.589	0.919	0.327	0.952
Splacn.	logNPH	−0.620	−0.753	0.602	0.929
	logBPL	−0.633	−0.835	0.509	0.956
	logZYB	−0.463	−0.245	0.936	0.936
	Eigenvalue	2.503	3.423	2.23	
	% Variance	99.98	57.06	37.17	
	Correlation	0.992			

**Note:**

The second column shows the loadings of the cranial variables on each block of the first dimension, the eigenvalue and the correlation between the scores on each block (neurocranium and splanchnocranium). The fifth column displays the communalities (h^2^) of each of the six variables retained in the two selected factors. The third and fourth columns show the loadings for the variables, the eigenvalues and the percentage of variance explained by the first two factors, respectively. GOL, glabella-opistocranion length; XCB, maximum biparietal cranial breadth; BBH, basion-bregma height. Splanchnocranial variables: BPL, basion-prosthion length; NPH, nasion-prosthion height; ZYB, bizygomatic breadth.

**Figure 3:**
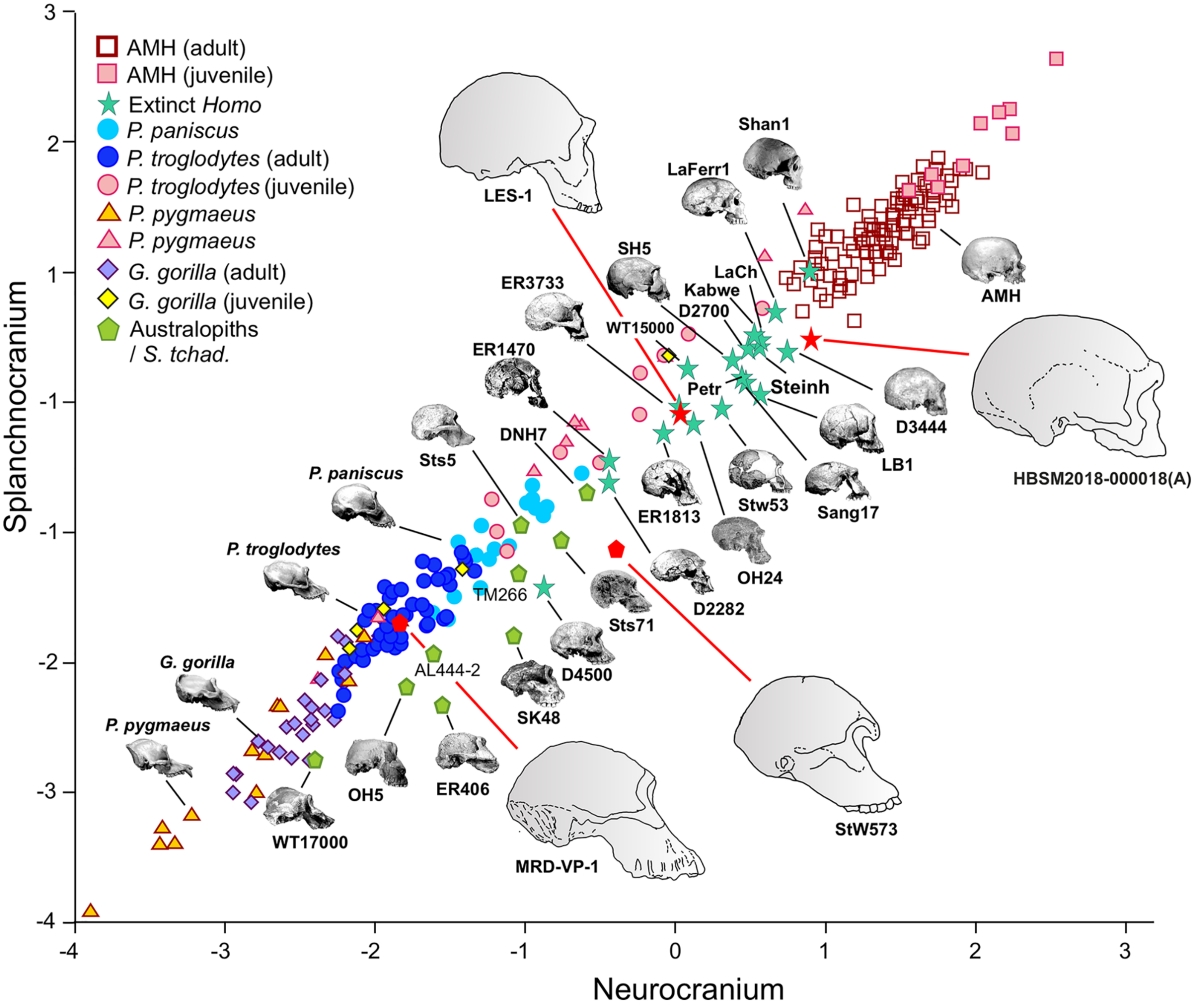
Non-pooled within-species partial least squares. 2B-PLS plot of the face *vs* neurocranium for size-scaled adults and juveniles of the great apes, modern humans and fossil specimens of extinct hominins. The pictures shown for the hominid and hominin crania are from Pérez-Claros, Jiménez-Arenas & Palmqvist (2015), with the exception of the four specimens depicted at a larger size and with red arrows, which were drawn by J.A. Pérez-Claros: cranium MRD-VP-1/1 of *Australopithecus anamensis* (based on Fig. 1d of Haile-Selassie et al., 2019); cranium StW 573 of *A. prometheus* (based on Fig. 5 of Clarke & Kuman, 2019); cranium HBSM2018-000018(A) of *Homo longi* (based on Fig. 1B of Ji et al., 2021 (license: CC BY-NC-ND 4.0)); and cranium LES1 of *H. naledi* (based on a screen shot from a 3D model available at https://www.morphosource.org/concern/media/000054666?locale=en).

In the case of 2B-PLS, the results are unidimensional and account for almost 100% of the original variance. This dimension combines in one block all the variables measured in the face (splanchnocranium), which all take high loading coefficients with negative sign, and those estimated in the neurocranium (the other block), which all show high and positive loadings. Therefore, the specimens that have a small neurocranium show a comparatively large face, while those with a well-developed neurocranium display a relatively smaller face (Fig. 3). Consequently, taking as a reference the skulls of the adult individuals of the extant species, a gradation is observed starting with the orangutan and continuing with the gorilla, chimpanzee, bonobo and, finally, the anatomically modern humans, which are separated from the rest by a morphological gap. This gap is filled by the extinct species of the genus *Homo*. The australopithecines are placed next to the great apes, although they show neurocrania of similar size with smaller splanchnocrania. Specimen D4500 from Dmanisi, which has a cranial capacity of only 546 cm^3^ (Rightmire et al., 2017), is the only one representative of the genus *Homo* that projects close to the australopithecines.

**Figure 4:**
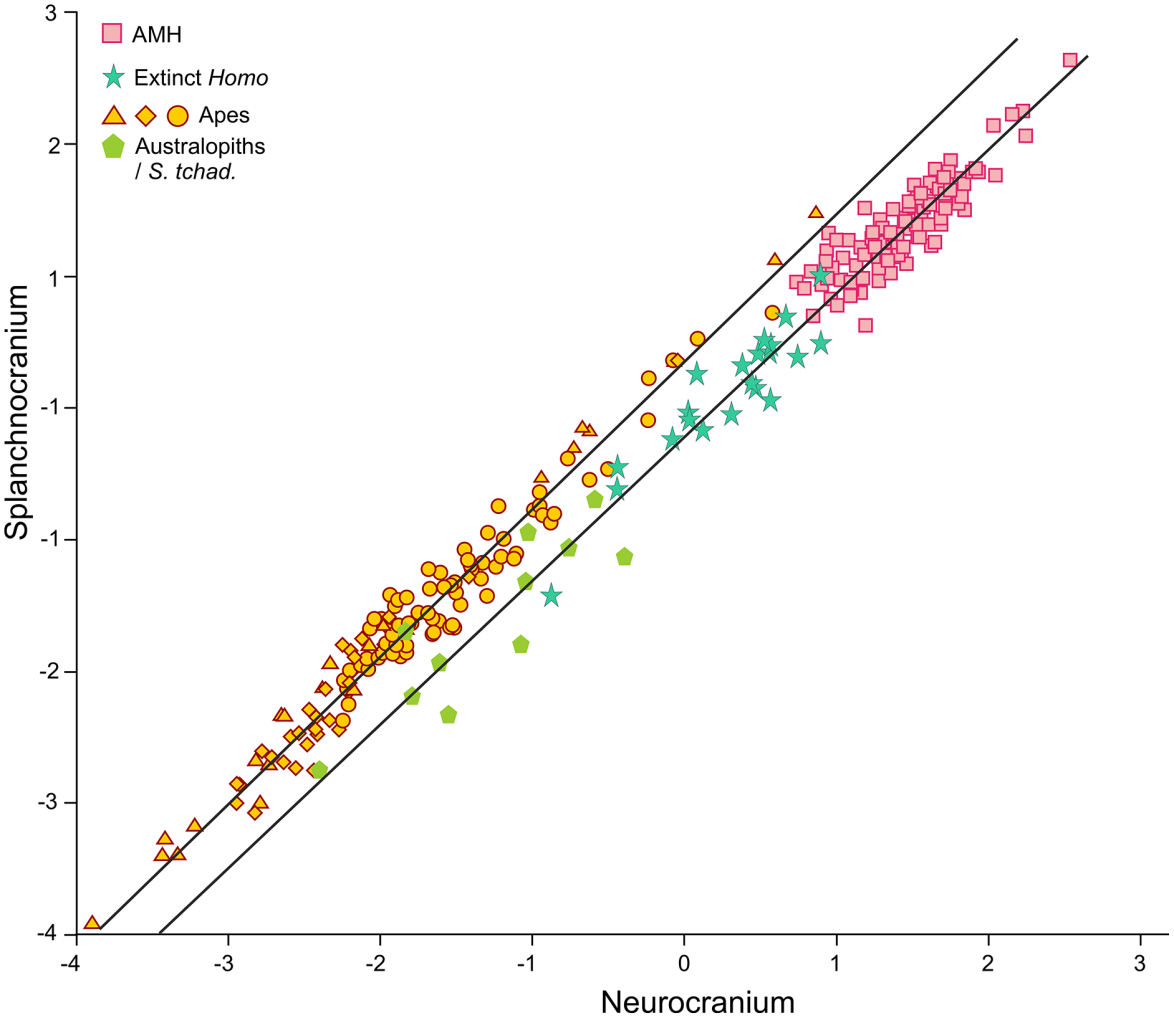
Non-pooled within-species 2B-PLS plots of the face *vs* neurocranium. This figure is the same as Fig. 3 and shows superimposed the fitted RMA lines for the great apes and the rest of the specimens analyzed. Note that both lines run in parallel and are distinguished by their intercepts. Consequently, for a given score on the neurocranial axis, the great apes show larger splanchnocrania than the australopithecines or the members of *Homo*.

Visual inspection of the arrangement of the new fossils included in this analysis allows us to tentatively make fruitful observations (Fig. 3). LES-1 is located very close to the earliest members of *Homo*, specimens ER-1813 and OH 24 of *H. habilis*, and also to specimen ER-3733 (the values of the latter and LES-1 are nearly identical). The two new specimens of *Australopithecus* included in the analysis (MRD-VP-1/1 and StW 573) score in the 2B-PLS morphospace next to the australopithecines, but in distinctly different positions (Fig. 3). It is important to take into account the two different trends of cranial shape and size shown by the great apes and the hominins (Fig. 4). The slopes of the reduced major axis (RMA) lines for the great apes and the hominins (1.112 and 1.084, respectively) are statistically indistinguishable (*p* = 0.111). However, the 95% confidence intervals of their intercepts (0.29735, 0.42694) and (−0.27133, −0.13696), respectively, do not overlap. This indicates that even in the case of those great apes with large neurocrania like the gorillas, they retain a relatively larger facial size than all the hominins, including the australopithecines. Cranium StW 573 of *A. prometheus* shows a projection that is closer to the RMA line of the hominins than to the one for the great apes. Moreover, it also has a ratio between the relative sizes of the neurocranium and the splanchnocranium that is among the highest of the australopithecines (Fig. 4). In contrast, cranium MRD-VP-1/1 of *A. anamensis* projects squarely onto the RMA line for the great apes, showing a ratio between the relative sizes of the modules that is similar to the one of specimen AL444-2 of *A. afarensis*, its hypothetical descendant species (Haile-Selassie, 2010; Haile-Selassie et al., 2019).

**Figure 5:**
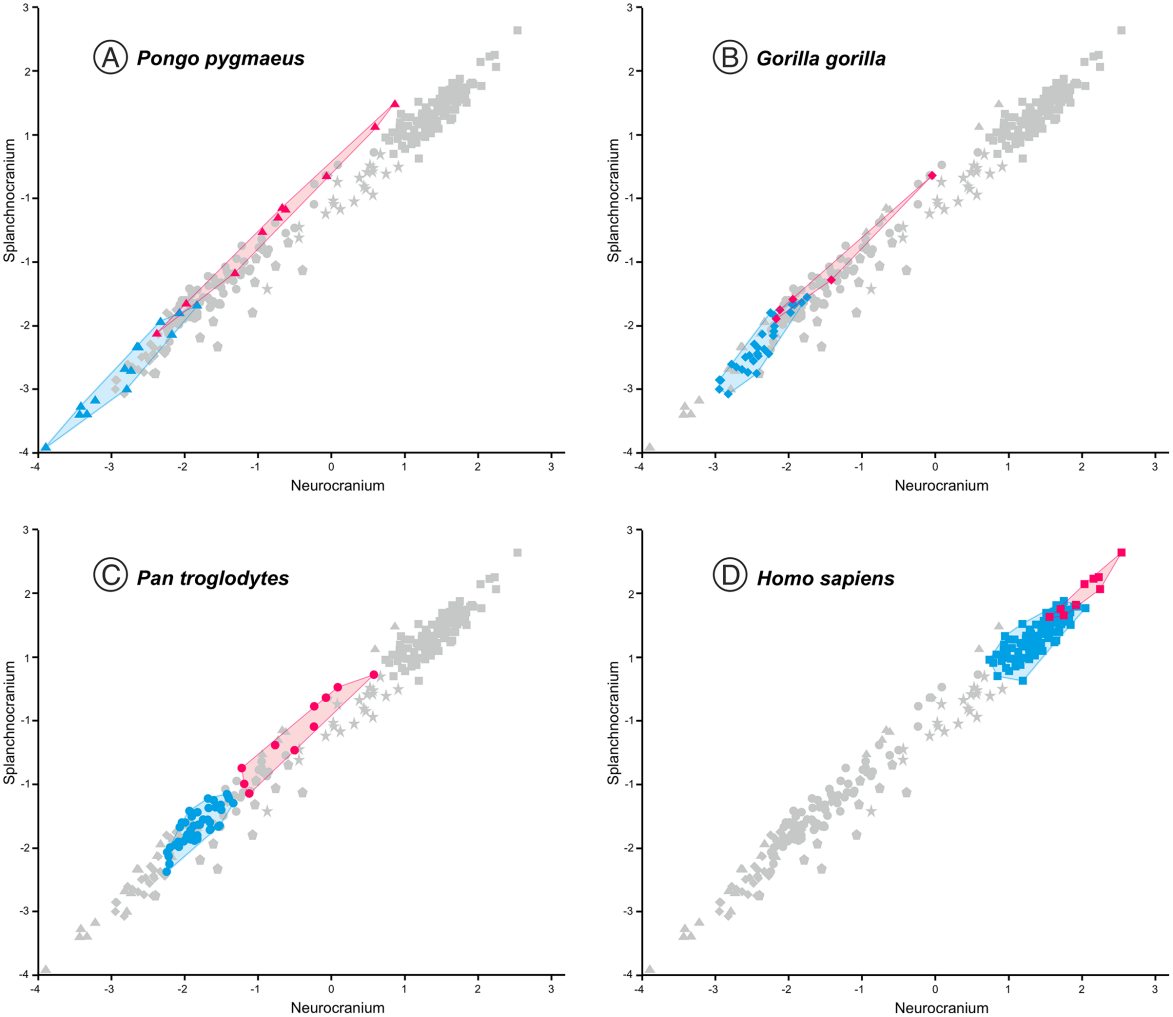
Non-pooled within-species 2B-PLS plots of the face *vs* neurocranium. This figure is the same as Fig. 3, but the convex hulls for the juveniles (pink tone) and adults (blue tone) of the extant species analyzed are showed separately. Note that in all cases the juvenile individuals show higher scores for the neurocranium. (A) orangutans. (B) gorillas. (C) common chimpanzees. (D) modern humans.

The position of the infants/juveniles of the extant species can be seen more clearly in Fig. 5. In all these individuals the ratio of neurocranial to facial size increases relative to their adults. Some sufficiently small skulls (which would presumably belong to the earliest ontogenetic stages) of the species of great apes may reach relative neurocranial sizes that are like those of the adult anatomically modern humans (AMH). However, they score along the great ape RMA line (Fig. 4). This indicates that, for a given neurocranial size, they show splanchnocrania that are slightly larger.

**Figure 6:**
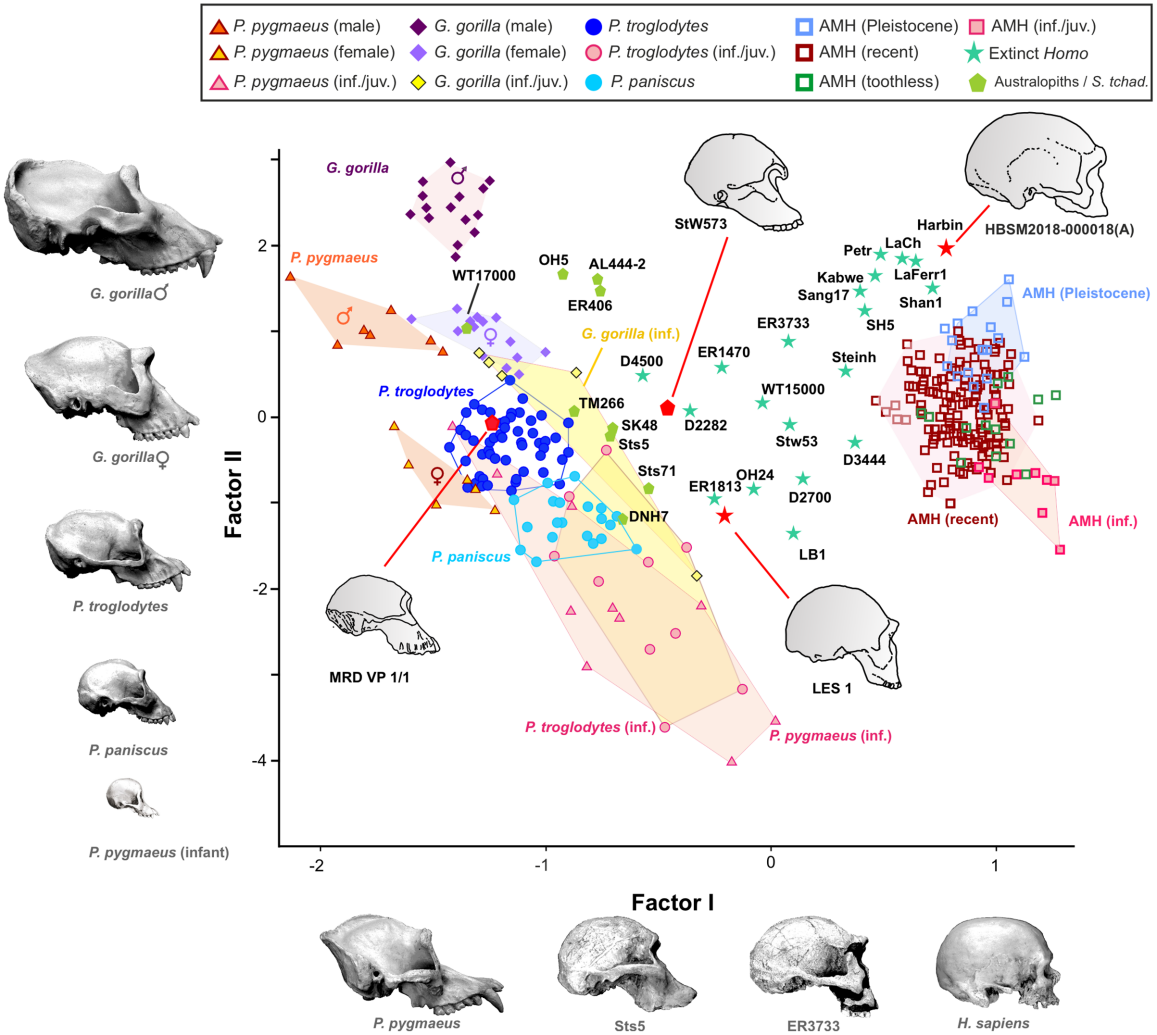
Factor analysis of the anthropometric variables used to estimate the length, width and height of each cranial module. The first factor (shape vector) explains 57% of the variance. Species are distributed along this axis according to the relative size of the neurocranium *vs* the splanchnocranium. The second factor relates to absolute cranial size and accounts for 37% of the remaining variance. The credits of the pictures and drawings of hominid crania appear in the legend of Fig. 3.

In contrast to the 2B-PLS analysis, which only considers shape differences among the crania compared, axes I and II of the factor analysis separate the shape and size components of cranial variation (Table 1, Fig. 6). As shown by Pérez-Claros, Jiménez-Arenas & Palmqvist (2015), in the case of the factor analysis presented here, any vector connecting two observations with equal shape (isometry) forms an angle of only 4.6° with factor II and of 85.4° with factor I. This indicates that it can be reasonably assumed that factor II can be basically interpreted as a size vector while factor I corresponds to a shape vector. The great apes and AMH score in the opposite regions of the first axis. In this axis, the variables measured on the neurocranium take positive loadings while those taken on the splanchnocranium load negatively. This again indicates that this axis can be interpreted in an *ad hoc* manner as a shape vector (Blackith & Reyment, 1971). The specimens analyzed are arranged along this axis in an increasing order of relative neurocranial size, with the fossil crania of extinct hominins occupying the intermediate region between the great apes and AMH. In contrast, all the variables measured in the neurocranium and splanchnocranium take positive loadings on axis II, which substantiates that it corresponds to a size vector.

The projections of the specimens on the morphospace depicted by axis I and II of the factor analysis (Fig. 6), which jointly account for more than 94% of the total variance (Table 1), are similar to those previously obtained by Pérez-Claros, Jiménez-Arenas & Palmqvist (2015). For this reason, we will focus on the new specimens analyzed here. LES-1 projects into the morphospace next to specimen KNM-ER 1813 (Fig. 6), showing features typical of the early members of *Homo*. The positions of the two newly described australopithecines indicate that they show opposite characteristics, as previously suggested by the 2B-PLS analysis (Figs. 3 and 4). StW 573 scores adjacent to the australopithecine group, in a close position to skull D2282 from Dmanisi. In fact, StW 573 shows a higher score on the first axis than D4500. In contrast, MRD-VP-1/1 projects on the opposite side of the morphospace, placed squarely within the common chimpanzees. Among the australopithecines, only KNM-WT 17000 falls within the region occupied by the great apes, in particular within female gorillas.

**Figure 7:**
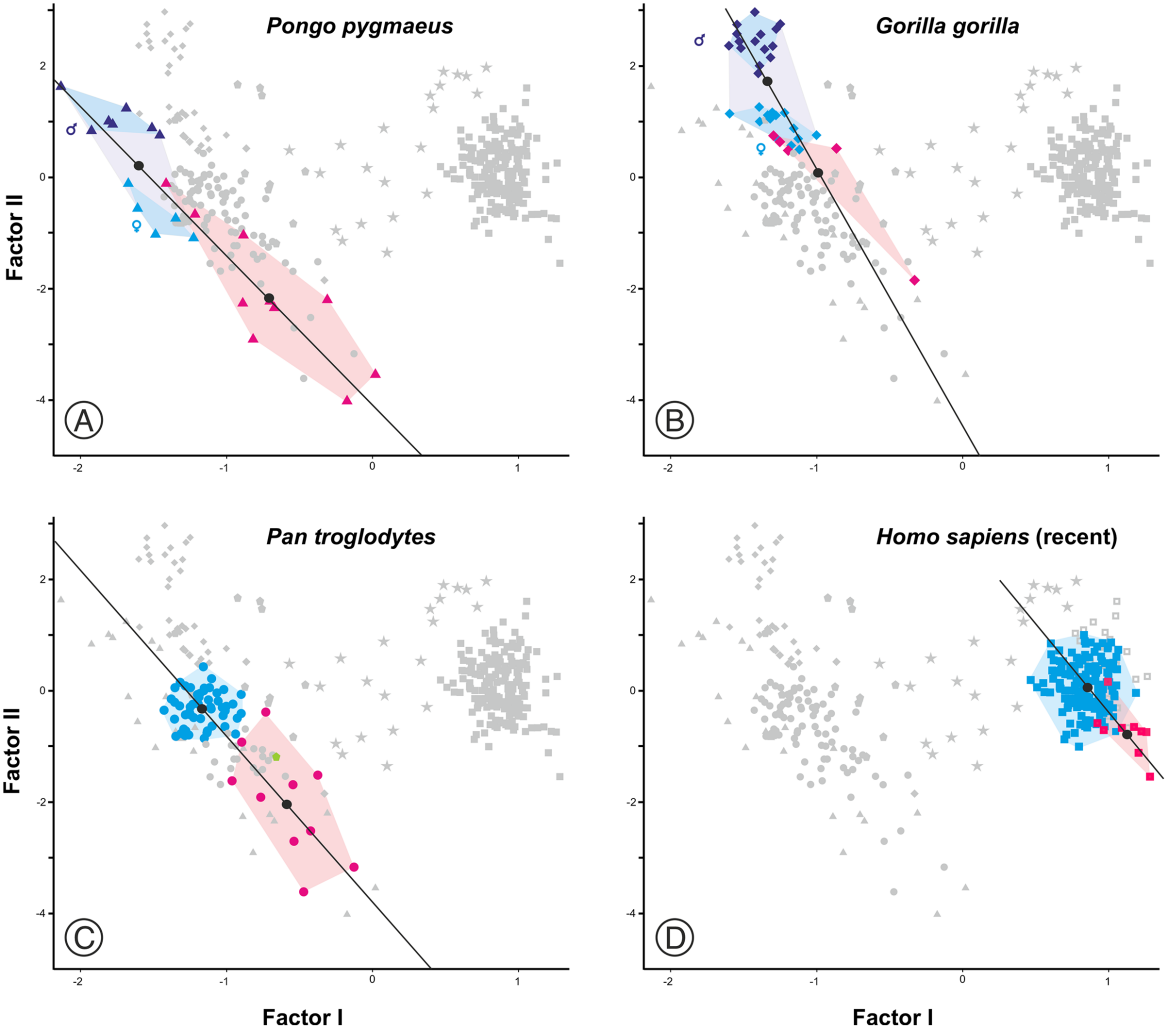
Plots of the factor analysis shown in Fig. 6 that highlight the convex hulls for adult and juvenile individuals of the extant species analyzed. (A) Orangutan. (B) Gorilla. (C) Common chimpanzee. (D) Modern humans. Pink symbols for juveniles and blue symbols for adults. In the case of the most dimorphic species (orangutan and gorilla), dark and light blue symbols correspond to males and females, respectively. The centroids of each group (black circles) are connected by lines.

**Figure 8:**
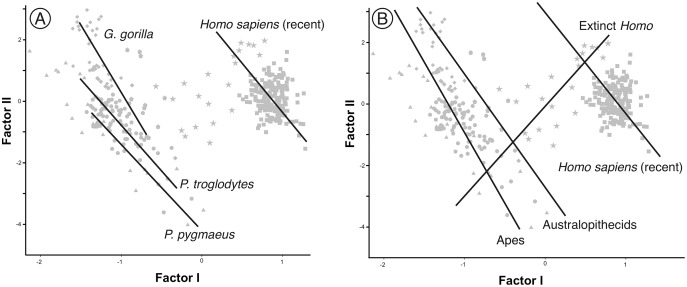
Factor analysis with RMA lines adjusted for different subsets. (A) Juveniles and adults of the extant species of hominoids. (B) Different sets of adult specimens. “Apes” refers to all adults of the analyzed species of great apes (*P. pygmaeus, G. gorilla, P. troglodytes*, and *P. paniscus*). “Australopithecines” refers to the analyzed specimens of *Australopithecus* and *Paranthropus*. Extinct *Homo* refers to all specimens of the genus *Homo* excluding AMH.

As expected, infants and juveniles of the living species show lower scores on factor II (largely a size vector) than their respective adults. However, given that as the cranium becomes smaller its neurocranium/face ratio also increases, a negative allometry is obtained in all cases. This ontogenetic allometry is more clearly evidenced in Figs. 7 and 8A. In the case of Fig. 7, the centroid of the infants/juveniles of each extant species has been joined with a straight line to that of their respective adults. The values of the angle formed by these lines with factor I for orangutans (158.8°), chimpanzees (161.1°) and humans (161.8°) do not differ significantly (especially in the case of chimpanzees and humans). Gorillas have a similar but somewhat larger angle (167.7°), but the sample analyzed is too small to obtain conclusive results (as mentioned earlier, they have only been retained in these analyses to show that they also display negative allometry). Therefore, Fig. 7 shows that the main difference between the extant species lies basically in the starting position along the horizontal axis (*i.e*., their Y-intercepts with Factor II). The RMA lines obtained for the extant species including adults and non-adults are shown in Fig. 8A. The slopes of such lines, together with other statistics, are presented in Table 2. All extant species show significant negative allometries and the differences among their slopes are not statistically significant, with the only exception of the gorilla (which differs statistically from the other three hominoids, *p* < 0.01 in all cases). As indicated above, the sample of gorillas is very small. In any case, these results indicate the existence of an ancestral negative allometry in hominids, which was retained in the hominins. Figure 8B shows the RMA lines, derived exclusively for the adult specimens of the great apes, the australopithecines, AMH and the extinct representatives of the *Homo*. Except for the fossil specimens of *Homo*, all slopes are negative and take relatively similar values (Table 2). It is noteworthy that if *A. anamensis* and *P. aethiopicus* (which both score close to the great apes) are excluded from the analysis, the value of the coefficient of correlation for the australopithecines becomes significant for *p* < 0.1. Such exclusion can be justified in the context of the ontogenetic scaling and lateral transpositions discussed below. In any case, the slope obtained for the australopithecines is not statistically different from those for the adult great apes (−4.508, *p* = 0.252) and AMH (*p* = 0.333). Moreover, the slope of the australopithecines is also statistically indistinguishable from those of the four living species shown in Fig. 8A (*p* > 0.250 in all cases). In contrast, the slope for the members of *Homo* excluding AMH is positive and differs statistically from those obtained for all the groups of specimens considered above, including AMH (*p* < 0.0001 in all cases).

**Table 2:** RMA slopes of the scores on factor II *vs* those on factor I for several sets of extant hominoids and extinct hominins analyzed.

Species	Slope	95% CI	r	*n*	P (r = 0)
*Pongo pygmaeus*	−2.820	[−3.239 to −2.282]	−0.918	24	<0.00001
*Gorilla gorilla*	−4.418	[−5.068 to −2.145]	−0.758	34	<0.00001
*Pan troglodytes*	−2.977	[−3.635 to −2.290]	−0.746	64	<0.00001
*Homo sapiens* (Recent)	−3.181	[−3.627 to −2.797]	−0.216	151	0.0077
Apes (Adults)	−4.534	[−5.291 to −3.601]	−0.569	117	<0.00001
Australopithecines	−3.671	[−10.718 to 2.647]	−0.346	10	0.3270
Extinct *Homo*	2.851	[1.887 to 3.610]	0.688	21	0.0006
AMH (Adults)	−3.203	[−10.124 to −2.787]	−0.015	142	0.8600

**Note:**

95% CI, *p* = 0.05 confidence intervals; r, coefficient of correlation; N, sample size; P, probability of r = 0.

**Figure 9:**
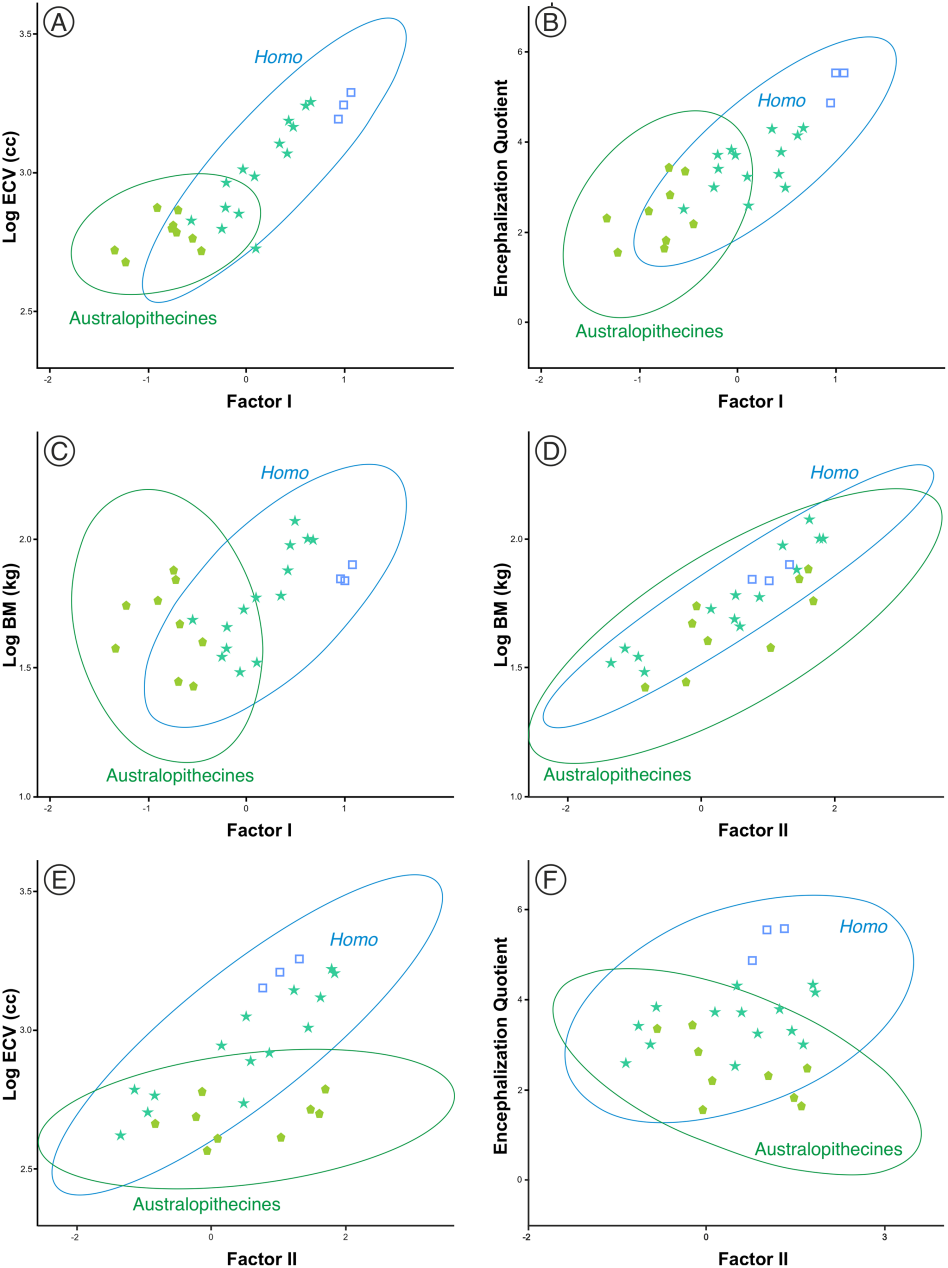
(A–F) Bivariate plots of different cranial variables on the scores on factors I and II. The data correspond to the sample of australopithecines and fossil *Homo* for which the variables necessary for the calculation of the encephalization quotient were available. ECV is endocranial volume (in cm^3^) and BM is body mass (in kg). The variables are Log_10_ transformed. All data and details of the calculation are provided in Table S2.

One aspect to consider with respect to the two sets of fossil hominins considered, the australopithecines and the members of *Homo*, is the relationship of the size and shape factors with body mass (BM), brain volume (ECV) and encephalization quotient (EQ). For this reason, a subset of them was selected (including three specimens of middle Pleistocene AMH) for which published values of such variables are available (Table S2). The correlations between these variables in both sets are shown in Table 3. The correlation of the variables with factor I are clearly different in the australopithecines and in the members of *Homo*. The correlations of factor I with body mass, brain volume and encephalization quotient are not significant for the australopithecines, but are significant for the fossil specimens of *Homo* (Figs. 9A–9C). In contrast, body mass and cranial size (Factor II) are correlated in both groups (Fig. 9D). Interestingly, endocranial volume does not correlate with skull size in the australopithecines, as opposed to *Homo* (Fig. 9E). Furthermore, cranial size is not related to the encephalization quotient for either the australopithecines or *Homo* (Fig. 9F). Given that there is a correlation in *Homo* between the scores on Factor I and geological age (r = 0.772, *p* < 0.001), it is pertinent to analyze whether this evolutionary trend was guided by the increase in brain size or in encephalization quotient. The partial correlation coefficient between Factor I and endocranial volume after controlling for EQ is statistically significant (r = 0.679, *p* < 0.005), but the partial correlation of Factor I and EQ after controlling for endocranial volume is not significant at 0.05 (r = 0.427, *p* = 0.099). This result suggests that the relative increase of the neurocranium with respect to the splanchnocranium in *Homo* relates more to the increase in the absolute size of the brain than to the increase in EQ value.

**Table 3:** Correlations between the scores on the first two factors, body mass, endocranial volume and encephalization quotient for the subsamples of australopithecines and members of the genus *Homo* analyzed.

		Factor I	Factor II	LogBM	LogECV
Australopithecines	Factor II	−0.330			
	LogBM	−0.199	**0.766**		
	LogECV	0.336	0.341	0.256	
	EQ	0.393	−0.595	**−0.840**	0.298
*Homo* (Fossil)	Factor II	**0.622**			
	LogBM	**0.695**	**0.938**		
	LogECV	**0.872**	**0.859**	**0.886**	
	EQ	**0.798**	0.480	0.377	**0.759**

**Note:**

Values in bold indicate significant correlations at *p* < 0.05.

## Discussion

The 2B-PLS analysis indicates that although the relative position of juveniles to adults within the extant species depicts a common pattern of covariation, the development of both cranial modules departs in the great apes from a more peramorphic starting point than in the rest of the hominoids analyzed. As a result, although showing parallel trajectories with some overlap, the relative size of the modules observed in most australopithecines or in the members of *Homo* cannot be reached from the developmental trajectory of the great apes. This points to a fundamental dissociation between the relative sizes of both cranial modules during the early stages of development in the hominins compared to the great apes. However, it is important to note that this must be interpreted from a statistical point of view, given that some hominins (*e.g*., *A. anamensis*) project close to the apes.

According to Lieberman & McCarthy (1999), cranial base angulation differs substantially between *H. sapiens* and *P. troglodytes:* While the human cranial base flexes postnatally in a rapid growth trajectory that is complete by 2 years, the cranial base in the chimpanzee extends postnatally in a more prolonged skeletal growth trajectory. Moreover, basicranial extension in non-human primates follows an extended growth trajectory, which mirrors the rate of growth of the face as a whole (Moore & Lavelle, 1974; Lieberman & McCarthy, 1999).

Factor analysis provides different and complementary information to that provided by 2B-PLS. Given that one of the axes obtained in the factor analysis of the specimens examined relates to the size of the specimens measured while the other accounts for the shape differences among them, the relationship between both axes determines a number of allometries that can be used for the characterization of heterochronies (Klingenberg, 1998; Smith, 2001; Vinicius & Lahr, 2003).

**Figure 10:**
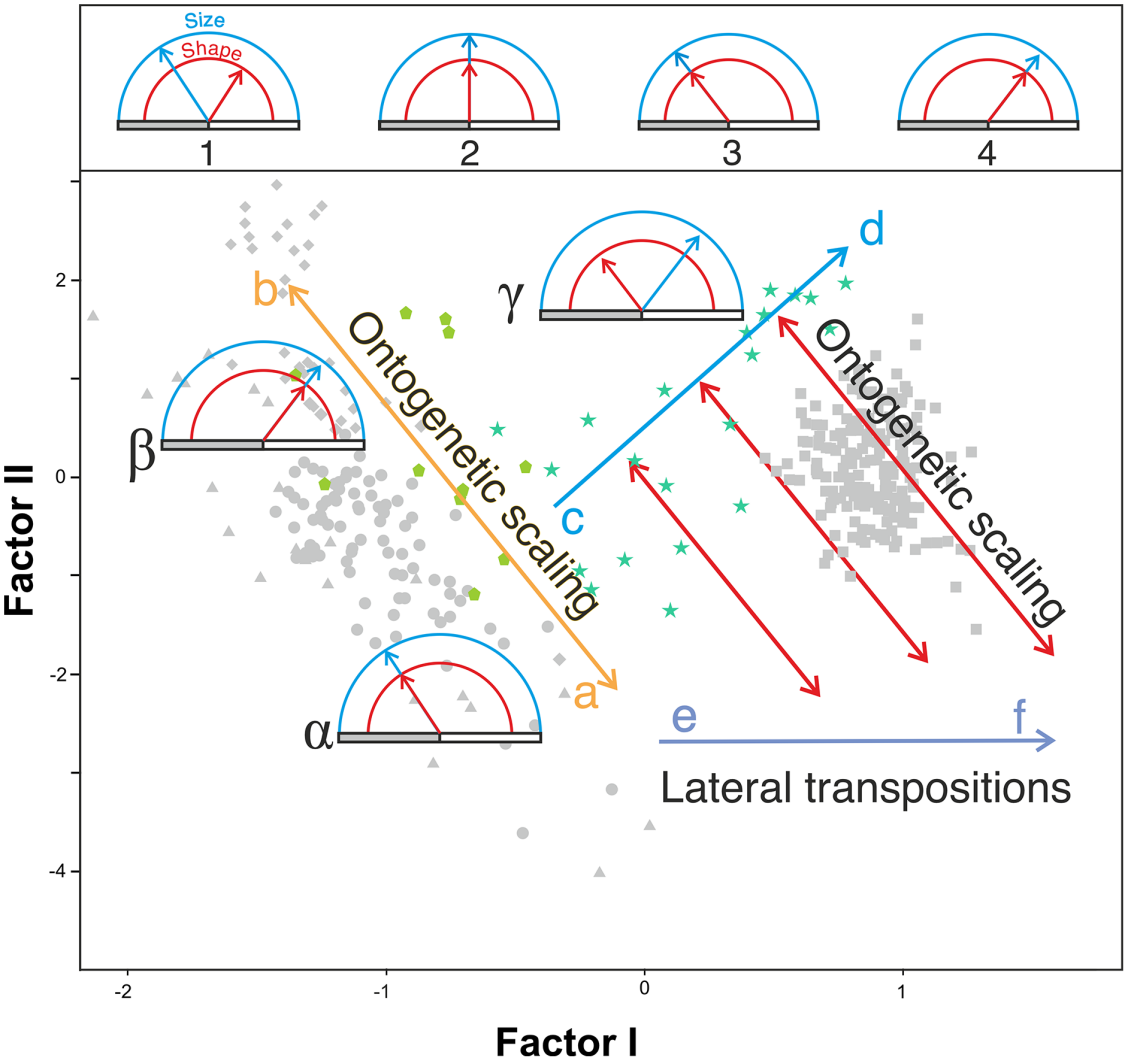
Proposed model of the allometric heterochronies in this study, illustrated with Gould’s clock model (Gould, 1977) on the plot of factor analysis shown in Fig. 5. The upper part of the figure shows different states of the clock, which indicates the change in size (blue) and shape (red) of a derived species with respect to an ancestral one. The horizontal bar is a marker of age. Ontogenetic development (of constant duration in these examples) begins at the left end of the gray bar and ends at its right end (adult state). Clock 1 indicates an arbitrary change in shape and size of a derived species with respect to a reference ancestral species shown in clock 2, which has been calibrated with both handles “at 12 o’clock”. In clock 3 and 4 the derived species retain the shape and size relationship of the ancestral species (clock 2) but their ontogenetic trajectories have been truncated or extended by ontogenetic scaling. See explanation in the text for the lower part of the figure.

In agreement with Gould (1977), the results obtained here highlight from a quantitative perspective the existence of an ancestral negative ontogenetic allometry between the neurocranium and the splanchnocranium, which is conserved in both the extant apes and modern humans. As expected, this fact is reflected in the existence of a shared pattern of integration in hominids (Mitteroecker & Bookstein, 2008; Singh et al., 2012; Scott et al., 2014; Neaux, 2017; Neaux et al., 2019). Therefore, to assume the existence of such allometry in the extinct hominins is a parsimonious hypothesis. However, this study also shows that there is another factor superimposed on the general allometric rule that describes the relationship between the neurocranium and the splanchnocranium: the shared allometries depicted by the different groups analyzed depart from different points. More specifically, for a given skull size the great apes have larger faces relative to the neurocranium than in the case of humans. This is clearly illustrated by comparing *P. troglodytes* and *H. sapiens* in Figs. 7A, 7B and 8B: both species show similar slope values, but differ in their intercepts. Our interpretation of the heterochronic consequences of such allometries are graphically depicted in Fig. 10.

Our model explains the results obtained combining two basic and independent types of evolutionary change in the ontogenetic trajectories: ontogenetic scaling and lateral transposition (Klingenberg, 1998). In the evolutionary trend displayed by *Homo*, these changes took place in a coordinated fashion. The distinction between both groups of processes may help to avoid much of the confusion and discrepancy that arises when different organisms are compared, as there does not seem to be a single heterochronic process that explains all cranial diversity observed among the living hominoids and the extinct hominins.

Ontogenetic scaling, considered as changes by extension or truncation of the ancestral trajectory (Klingenberg, 1998), seems pervasive in the species analyzed. Traditionally, this process has been proposed, at least in part, as a mechanism of evolutionary change in primates (Giles, 1956; Pilbeam & Gould, 1974; Shea, 1983, 1985; O’Higgins & Collard, 2002; Berge & Penin, 2004; Viϑarsdóttir & Cobb, 2004). In the case of those aspects analyzed in this article, it is associated with the ontogenetic allometry mentioned above, which is retained in hominins. The African apes are a good example of this, since the ratio of face size to neurocranial size of bonobos, chimpanzees and gorillas follows the same allometric trajectory of increase with cranium size. If ontogenetic scaling is not appreciably altered by other changes in growth trajectory, peramorphosis and paedomorphosis can be established directly and unambiguously (Klingenberg, 1998). Therefore, bonobos are paedomorphic in the traits analyzed with respect to chimpanzees and the latter are paedomorphic compared to gorillas (Shea, 1988). It is important to note that paedomorphosis and peramorphosis in the present study are used exclusively in terms of a result and do not refer to a specific process. Consequently, in the approach used here, the same followed by Gould (1977) and Shea (1989), there is a clear separation between the underlying biological processes and the final morphological results. This implies that comparable or identical morphologies can be obtained by different ontogenetic processes (Shea, 1989). In the same line of reasoning, Klingenberg (1998) pointed out that ontogenetic scaling can be the result of progenesis (acceleration in gonadal development, which leads to precocious offset) and hypermorphosis (delayed onset of sexual maturity), but also of changes in growth rates or onset time. Ontogenetic scaling is illustrated in Fig. 10 with Gould’s clock model (Gould, 1977) and using as examples two heterochronic phenomena defined by Shea (1983): rate hypomorphosis (Fig. 10, α) and rate hypermorphosis (Fig. 10, β). The crania located close to the orange line a-b of Fig. 10 can be considered paedomorphic with respect to a reference form located at the center if their position along this line is closer to the “a” end and peramorphic if they are closer to the “b” end, respectively. It is important to note that the sets of species that can be grouped under the same process of allometric scaling are those for which a linear extension of allometry in the growth direction can be established. For example, AMH cannot be considered as part of the ontogenetic scaling that groups the African apes, because they are laterally displaced with respect to them. Therefore, to share the same ancestral ontogenetic allometry does not imply the same process of ontogenetic scaling. It is important to note that the model proposed here implies that any two specimens compared in this morphospace differ in two independent aspects: the degree of progression along their respective ontogenetic scaling and the distance that separates them resulting from lateral transposition. Pérez-Claros, Jiménez-Arenas & Palmqvist (2015) proposed three groups of extant hominids and extinct hominins according to distinct interspecific allometries within the morphospace analyzed in the present study. These groups can be taken as a reference for defining independent processes of ontogenetic scaling: (i) the African apes; (ii) the australopithecines; and (iii) AMH together with *H. heidelbergensis* and *H. neanderthalensis*, to which *H. longi* can be incorporated (Fig. 6). According to the model proposed here, these three sets are separated by lateral transpositions, which indicate that the starting forms differ between the ancestor and the descendant (Klingenberg, 1998). Lateral transpositions involve a decoupling of the growth trajectories of traits in the early stages of development (Klingenberg, 1998).

Figure 10 shows the model proposed here for the evolution of the cranium in the case of the genus *Homo* (line c-d). This model can be decomposed in two sets of processes that occurred in a coordinated fashion: extension of ontogenetic scaling (red lines) coupled to lateral transpositions along the violet line e-f. The paradox is that although ontogenetic scaling makes the shape of the skulls more peramorphic with respect to earlier ontogenetic stages (according to the ancestral ontogenetic allometry), lateral displacements make it more paedomorphic. The result is shown in Fig. 10 (γ) with Gould’s clock model for the heterochrony of *Homo*: cranial size increases while cranial shape is progressively more paedomorphic. Unlike the australopithecines, this phenomenon resulted in an increase of brain volume (as measured in the endocasts) with increasing cranium size in the genus *Homo* (Fig. 9E). Since shape (Factor I) correlates with cranial size (Factor II) in the genus *Homo* (but not in the great apes), the result is that both endocranial volume and the encephalization quotient increase also with the ratio between neurocranial and facial size. In the australopithecines such relationship is not observed (Figs. 9A and 9B). In other words, increasing cranial size as in an australopithecine does not result in an appreciable increase in endocranial volume (Fig. 9E). Given the existence of a pervasive negative allometry between the neurocranium and the splanchnocranium through the ontogenetic development of all hominids, possibly the most feasible evolutionarily mechanism that led to the increase in brain size in the genus *Homo* was a pervasive dissociation of the growth patterns of both cranial modules through lateral transpositions. This allows us to interpret that an increase in the starting size of the neurocranium was likely the most feasible mechanism that allowed a larger endocranial volume at the end of growth. Given that adult brain volume is highly correlated with neonatal brain volume (DeSilva & Lesnik, 2008) the increase in brain size at earlier and earlier stages of development was reflected in the appearance of the “obstetrical dilemma” (for a thorough review on this issue, see Haeusler et al., 2021).

Given the constructional imposition of a negative ontogenetic allometry between the two cranial modules, successive lateral transpositions in *Homo* led both to the acquisition of larger neurocrania and to increased encephalization. At this point, the question that arises is whether the former or the latter of such acquisitions is behind the evolutionary trend in *Homo*. Recent research has pointed out that our cognitive abilities are not associated with the encephalization quotient but with the absolute number of neurons of the brain and, particularly, of the cerebral cortex (Roth & Dicke, 2005; Deaner et al., 2007), which is proportional to absolute brain size (Herculano-Houzel, 2009, 2012). The results obtained here seem to support that hypothesis, because the partial correlation of the degree of paedomorphosis with encephalization is not significant if absolute brain size is held constant. In other words, the higher degree of paedomorphosis in humans compared to the great apes is directly correlated with an increase in cognitive abilities. On the other hand, given that the brain is an energetically costly tissue, representing 20–25% of metabolic resting rate, the increase in brain size during human evolution also posed a challenge for metabolic expenditure. The rising metabolic demands were ultimately compensated by an increase in net energy input from a shift to an increasingly carnivorous diet, which has been associated with a reduction in the size of the gut (Aiello & Wheeler, 1995; Herculano-Houzel, 2012). However, it was the large brain itself that enabled the increased energy input through increased cognitive abilities. These abilities ultimately led to a diet of improved quality with a higher proportion of meat and cooked food (Aiello & Wheeler, 1995) as well as to new strategies based on social skills such as cooperative breeding and feeding (Antón, Potts & Aiello, 2014).

Another issue that arises from our model is the hypothetical possibility that face reduction is an associated effect of absolute brain enlargement and the cultural and technological development that the latter entailed. In fact, given that the size of the face relates to the size and arrangement of the dentition and its associated muscles, the reduction of the face in *Homo* may be related to an increase in cognitive abilities. This in turn allowed a higher degree of extra-oral food processing, either mechanically assisted by lithic industries (Zink & Lieberman, 2016) and/or by cooking (Wrangham & Carmody, 2010; Veneziano et al., 2018, 2019). A reduction in gut size also led to metabolic savings that could be used, among other destinations, to increase brain size. Accordingly, the neurocranial expansion and facial retraction that is usually interpreted in the evolution of the genus *Homo* as the result of strong directional selection (Schroeder & von Cramon-Taubadel, 2017) may have been the consequence of a positive feedback loop. In effect, natural selection may have promoted a feedback mechanism, which would be behind the evolutionary trend observed in *Homo*: increased cognitive abilities through the acquisition of a larger brain would allow the access to more energetic dietary resources, which would in turn enable the development of larger brains in parallel to the further increase in cognitive abilities that this entails. In other words, an increase in cognitive abilities can be both the cause and the effect underlying the evolutionary trend observed in our genus.

The trend towards increasing neurocranium size *via* successive lateral transpositions began with the first members of the genus *Homo* and continued until the Late Pleistocene. However, there are two exceptions, *Homo floresiensis and H. naledi*, which share a couple of characteristics: (i) they are the smallest of the genus *Homo* in the sample of adult crania analyzed; and (ii) both show proportions between the neurocranium and the face that are close to the habilines despite the fact that they are relatively recent (18 and 286 kyrs, respectively). A question that arises here concerns how both forms can be integrated into the proposed model of heterochronic changes. If they derived from ancestors with habiline-like cranial proportions, it is only necessary to allude to ontogenetic scaling. On the contrary, if they come from ancestors with larger neurocrania, lateral transpositions must also be involved. The latter possibility seems to be the case of *H. naledi* (endocast volume: 465–560 cm^3^ (Berger et al., 2015)), which has been considered as a miniaturized *H. erectus* (Schroeder et al., 2017). In the case of *H. floresiensis* (endocast volume: 426 cm^3^ (Kubo, Kono & Kaifu, 2013)), insular dwarfism from Asian *H. erectus* was also initially proposed, but this species is now considered as a habiline-related form (Argue et al., 2017; Parins-Fukuchi et al., 2019). Therefore, the relative size of both cranial modules in *H. floresiensis* would result from ontogenetic scaling from an ancestor close to *H. habilis*. The extreme reduction of brain size in *H. floresiensis* can be explained in terms of biological efficiency, given that a smaller brain implies a lower metabolic consumption, which may have had adaptive significance under conditions of insularity (Weston & Lister, 2009).

In contrast to the two species discussed, *Homo longi*, the recently described species of our genus (Ji et al., 2021), culminates the trend previously described in *Homo* towards a more paedomorphic form and larger size. From an evolutionary perspective, it seems to be an extension of the evolutionary trend of the genus *Homo* that continued after *H. neanderthalensis*. However, from an allometric point of view, *H. longi* is related to *H. sapiens* by ontogenetic scaling. Like *H. neanderthalesis*, *H. longi* can be conceived as a peramorphic form with respect to *H. sapiens*. As mentioned above, perhaps the skull of *H. longi* corresponds to that of a Denisovan (Gibbons, 2021). Genetic evidence indicates that Denisovans are closer to Neanderthals than to anatomically modern humans, although interbreeding between the three lineages has been documented (Slon et al., 2018; Rogers, Harris & Achenbach, 2020). Regardless of its specific taxonomic attribution, these results show that the cranium ascribed to *H. longi* shows distinctive characteristics. In any case, these cranial differences could result from genetic drift, as proposed for Neanderthals and modern humans by Weaver, Roseman & Stringer (2007).

In the case of the australopithecines, the proportions between their cranial modules follow the rule of ontogenetic scaling and do not obey any particular evolutionary trend to the extent that there is no increase or decrease of such a ratio over geologic time (Pérez-Claros, Jiménez-Arenas & Palmqvist, 2015). However, the two oldest species analyzed, *A. anamensis* and *A. prometheus*, show very different values in factor I (shape), as the former is projected within the great apes and the latter close to the habilines. In these cases, it is necessary to allude to lateral transpositions, which have also played an evolutionary role within the australopithecines. In the case of *A. anamensis*, it is commonly accepted as the ancestor of *A. afarensis* (Haile-Selassie, 2010; Haile-Selassie et al., 2019). Both species are laterally displaced on shape. Conversely, *A. anamensis* could be allometrically related to *P. aethiopicus*, which also projects into the area occupied by the great apes. Similarly, *P. aethiopicus* is considered a basal form of the genus *Paranthropus* (Kimbel, White & Johanson, 1988; Dembo et al., 2015; Mongle, Strait & Grine, 2019; Parins-Fukuchi et al., 2019) and later forms such as *P. boisei* are laterally displaced relative to the former in our analyses. In conclusion, ontogenetic scaling and lateral transpositions have been involved also in the allometries displayed by the australopithecines, but unlike the genus *Homo*, both phenomena do not occur in a coordinated manner, giving rise to a single evolutionary trajectory.

## Concluding remarks

This study focuses on the evolutionary change of allometries in the relative size of the neurocranium and splanchnocranium in the living hominids and extinct hominins. Our results show that the great apes and modern humans share a negative ontogenetic allometry in the neurocranium and a positive one in the splanchnocranium, which allows us to interpret the cranial heterochronies in terms of ontogenetic scaling processes and lateral transpositions.

For a given size of the neurocranium, scaling the crania to the same size shows that the great apes display larger splanchnocrania than the hominins. Given that the relative size of the splanchnocranium increases through the ontogeny, this results in more peramorphic skulls in the great apes.

The negative ontogenetic allometry between the neurocranium and the splanchnocranium allows us to establish two main groups of heterochronies. On the one hand, those based on ontogenetic scaling by extension (peramorphosis) or truncation (paedomorphosis) of an ancestral ontogenetic trajectory, as happens in the African great apes, the australopithecines, *H. sapiens*, *H. neanderthalensis* and *H. longi*. On the other, another source of heterochronies is based on lateral transpositions that imply a change in the starting relationship between the sizes of the neurocranium and the splanchnocranium at earlier ontogenetic stages. In the case of *Homo*, both types of processes have been combined in a trend towards larger paedomorphic crania.

## Supplemental Information

10.7717/peerj.13991/supp-1 Supplemental Information 1 Data used for this study. GOL: glabella-opistocranion length. XCB: maximum biparietal cranial breadth. BBH: basion-bregma height. BPL: basion-prosthion length. NPH: nasion-prosthion height. ZYB: bizygomatic breadth. All measurements are in mm. * indicates measurement taken on photographs for the new specimens included in the database. Bold type indicates measurement taken from 3D models.

10.7717/peerj.13991/supp-2 Supplemental Information 2 Subset of specimens analyzed with published estimates of body mass and endocranial volume. Estimates of body mass (BM) and endocranial volume (ECV) in kilograms and cubic centimeters, respectively.

## Additional Information and Declarations

### Data Availability

The following information was supplied regarding data availability: The raw measurements are available in the Supplemental Files.
